# Subacute Sclerosing Panencephalitis in a Child Suffering from Human Immunodeficiency Virus on “Highly Active Antiretroviral Therapy” – Can This be Another Instance of Immune Reconstitution Inflammatory Syndrome?

**DOI:** 10.7759/cureus.1346

**Published:** 2017-06-13

**Authors:** Ashutosh Gupta, Suman Kushwaha, Mushbiq Manzoor, Shah Faisal Ahmad Tarfarosh

**Affiliations:** 1 Senior Resident, Department of Neurology, Institute of Human Behaviour and Allied Sciences (IHBAS), Delhi, India; 2 Associate Professor & HOD, Department of Neurology, Institute of Human Behaviour and Allied Sciences (IHBAS), Delhi, India; 3 Resident, Department of Neurology, Institute of Human Behaviour and Allied Sciences (IHBAS), Delhi, India

**Keywords:** hiv, immune reconstitution inflammatory syndrome, sspe, highly active antiretroviral therapy (haart), neurology

## Abstract

We report a 12-year-old boy with human immunodeficiency virus (HIV) who presented with rapidly progressive difficulty in ambulation. The symptoms started to worsen when he was put on antiretroviral therapy (ART). Our findings show that the dynamics of HIV-related immune suppression and highly active antiretroviral therapy (HAART) have an impact on the clinical course of Subacute sclerosing panencephalitis (SSPE). Slow progression is expected in children on HAART but in our case, we observe a complex interaction of the virus with the immune system and modification of disease course of SSPE with ART. The child we discuss in this case report developed rapidly progressive SSPE on HAART regime; so the possibility of SSPE to be labeled as immune reconstitution inflammatory syndrome (IRIS) should be considered.

## Introduction

Subacute sclerosing panencephalitis (SSPE) is a devastating, progressive, destructive process of the central nervous system caused by the mutant measles virus infection of neurons [1]. Classical SSPE, first described by Dawson in 1934, is caused by latent measles virus 6-8 years after the primary measles infection [2]. However, early onset of SSPE with a fulminant course has been described in children infected with human immunodeficiency virus (HIV), as well as those perinatally infected with measles virus, probably due to lack of maternal antibodies and impaired host immunity [3-4]. The dynamics of HIV-related immune suppression and highly active antiretroviral therapy (HAART) have an impact on the clinical course of SSPE. SSPE is a rare neurological disease of childhood or young adulthood. The first signs are usually behavioral changes such as failing schoolwork, memory loss, and/or irritability. Involuntary muscle movements (myoclonic jerks) and generalized seizures follow. SSPE is a progressive disease which results in personality changes, outbursts of temper, sleeplessness, disorientation, stupor, spasticity, loss of previously acquired intellectual skills, poor memory and judgment (dementia), and general neurological deterioration. Blindness may develop because of a lesion in the vision center of the brain (cortical blindness) and the nerves of the eyes may waste away (optic atrophy). The late symptoms of SSPE may include muscle rigidity, elevated body temperature (hyperthermia) and/or abnormalities of respiration, heartbeat, and blood pressure. Slow progression can be expected in children on HAART. SSPE is a slow viral disease caused by altered measles virus with a progressive course [5]. There are not a great number of reports of SSPE in HIV-infected children in describing its progression. Complex interactions of the virus with the immune system and modification of its course with ART are still being studied. Here we report a case of SSPE in a child with HIV infection on ART. The disease course was fulminant unlike that of classical SSPE. Such cases have not been reported greatly in literature.

## Case presentation

A 12-year-old boy with acquired immunodeficiency syndrome (AIDS) diagnosed at 9 years of age and on ART for the last three years, presented with a history of gradual onset of rapidly progressive difficulty in ambulation for the past six months. The child subsequently walked with support and then progressively lost ability to sit or to use his limbs for activities of daily living. The patient became dependent on family members for all his activities. This was also associated with the reduction in verbal output for the same duration, which was limited to bisyllables at presentation. He was studying in 7th standard and developed declining in scholastic performance.

The patient gave a history of non-rhythmic, brief, involuntary and sudden jerks, involving upper limbs followed by lower limbs leading to dropping of objects for the past three months. This initially used to happen for four to five times a day but its frequency gradually increased and later happened once in every 10-15 seconds. These jerks used to persist in sleep. No other tonic-clonic or focal seizures were reported. There was no loss of consciousness, bladder or bowel symptoms, or visual deterioration.

He is the youngest of five siblings born out of a non-consanguineous seropositive couple with a normal birth and development history until 11 years of age. He had an exanthematous fever at 5 years of age, suggestive of measles which resolved without any immediate complications. The child was diagnosed with HIV at 9 years of age, one year after his parents were diagnosed. There was no prior history of blood transfusions or sexual abuse. The possibility of perinatally acquired infection was considered as perinatally acquired HIV infection present with symptoms only in adolescence and early adulthood as has been described in the literature. His CD4 count was 204 before initiation of ART. This child was initiated on triple-drug ART regime – Zidovudine, Nevirapine, and Lamivudine which was well tolerated. CD4 count increased to 419 before child developed neurological symptoms. Clinically, at presentation, his World Health Organization (WHO) staging for HIV virus was stage 2.

On examination, the child was conscious, alert, and oriented with normal vital parameters. There were multiple healed scars evident on his forehead and scalp, attributed to injuries sustained after dropping attacks. A cranial nerve examination, including the fundus, was unremarkable. Motor examination revealed 3/5 power in all four limbs and normal tone with normal deep tendon reflexes. No meningeal signs, involuntary movements, or cerebellar signs were evident. His gait and posture could not be tested as he was not able to stand without support.

**Figure 1:**
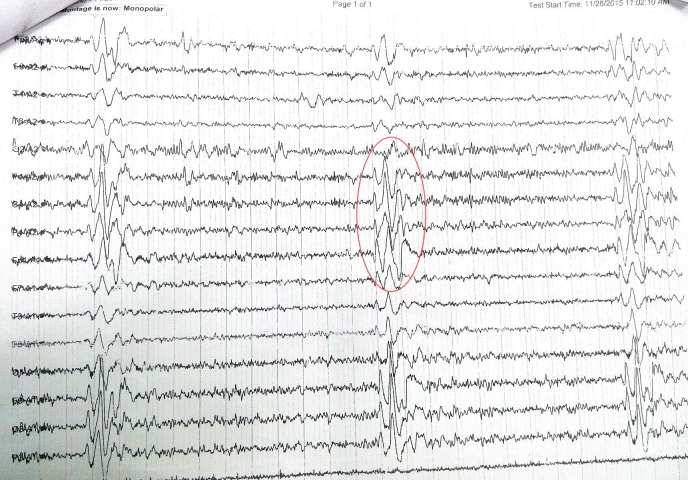
Electroencephalogram (EEG) revealed a slow background with periodic discharges suggestive of Radermecker complexes, recurring at regular intervals of every 8-10 seconds.

**Figure 2:**
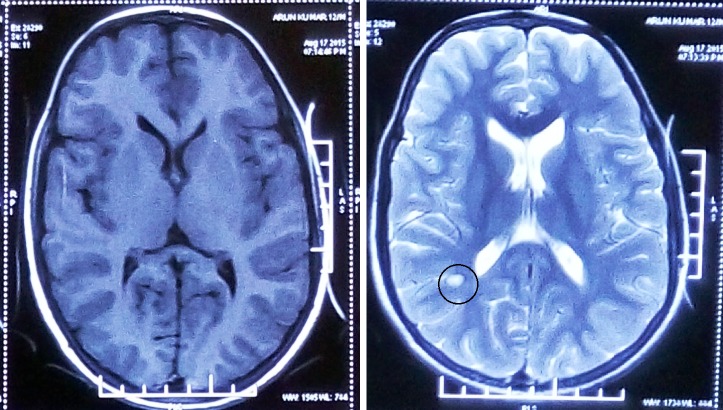
T1-weighted magnetic resonance imaging (MRI) sequences are grossly normal while T2-weighted MRI sequences showing nonspecific hyperintensities (encircled).

Laboratory evaluation revealed normal complete blood counts and normal renal and liver parameters. His CD4 count was 429. Electroencephalography of monopolar montage revealed generalized pseudo-periodic discharges at an interval of nine to 12 seconds (Figure 1). Cerebrospinal fluid cytology, glucose levels, and protein levels were normal. The measurement of specific antibodies by enzyme-linked immunosorbent assay revealed that measles immunoglobulin G antibodies were markedly elevated in the cerebrospinal fluid at 1:625 (normal range, <1:5). Magnetic resonance imaging revealed enhancements of affected regions in the early course of the disease in T1-weighted scan. T2-weighted scans show nonspecific hyperintensities and fluid attenuated inversion recovery sequences (Figure 2).

Hence the clinical picture, electroencephalogram, and cerebrospinal fluid patterns were pathognomonic for a diagnosis of SSPE.

The child received sodium valproate and clonazepam, after which the frequency of the child’s drop attacks initially abated, but later increased again. Other treatment options such as interferon and Isoprinosine were not considered because of financial constraints. The patient was on follow-up for two months but did not show any significant improvement.

## Discussion

The implications of the global HIV epidemic for measles-associated neurological disorders, immunological interactions between measles virus (MV) and HIV, and the role of vaccination in prevention are yet to be completely elucidated. To our knowledge, there are scarce reports of SSPE in HIV-infected individuals, and the natural history remains uncertain [6]. Neither the biology underlying the viral persistence nor the triggering mechanism for viral reactivation is well understood. The classic periodic complexes as described by Cobb [7] occur every four to 15 seconds. In our patient, it is occurring at eight to 10 seconds again emphasizing that our patient has SSPE.

Typically, affected persons have a history of measles infection in infancy [8], with clinical features of SSPE developing years later. Although decline may be punctuated by periods of clinical plateau, a gradual progressive course leading to death is invariable. Our patient also had history suggestive of measles.

The diagnosis of SSPE was confirmed by the detection of cerebrospinal fluid measles antibodies. The defective lymphocyte helper cells Th1 and its cytokines IFN and IL2 lead to persistence of altered MV virus and subsequent development of SSPE [9]. The level of impairment of Th1 cell function might dictate the clinical course. However, this area needs further research into the complex interaction of the altered virus and the role of HAART in containing the progression of the disease.

It should also be kept in mind that in any child with AIDS and presenting with myoclonic jerks, SSPE should be considered in addition to other causes. Our child was unvaccinated. We strongly recommend vaccination against measles to all specially in HIV cases due to the growing prevalence of case reports of fulminant SSPE with HIV positive patients. Measles vaccination is recommended as early as six months of life in HIV-infected infants as they are more likely to acquire measles before nine months of age and may not yet be severely immunocompromised at six months. Among HIV-infected children pre-HAART, monovalent measles vaccine at six months was well tolerated and immunogenic. These data support the current WHO recommendation to administer the first dose of measles vaccine at six months of age to HIV-infected children [10].

## Conclusions

The presentation of SSPE in HIV can both be classical fulminant progression or a slow progressive disease. Also it needs to be emphasized that as reported in few of the earlier cases and our case, the child developed SSPE while being on HAART regime; so possibility of SSPE to be labeled as IRIS should be considered in this case because when the immune system begins to recover, the previously acquired opportunistic infection, measles in this case, evokes an overwhelming inflammatory response that paradoxically makes the symptoms of infection worse. It has been shown earlier that children not immunized against measles had a significant rapid course of the disease. The same is true for our patient with HIV who deteriorated rapidly due to SSPE when he was put on ART.
